# Frequency distribution analysis of activation times and regional fibrosis in murine *Scn5a*^*+/−*^ hearts: The effects of ageing and sex

**DOI:** 10.1016/j.mad.2012.07.006

**Published:** 2012-09

**Authors:** Kamalan Jeevaratnam, Rebecca Rewbury, Yanmin Zhang, Laila Guzadhur, Andrew A. Grace, Ming Lei, Christopher L.-H. Huang

**Affiliations:** a Physiological Laboratory, University of Cambridge, Downing Street, Cambridge CB2 3EG, United Kingdom; b Cardiovascular Biology Group, Department of Biochemistry, Hopkins Building, University of Cambridge, Cambridge CB2 1QW, United Kingdom; c Department of Paediatrics, First Affiliated Hospital, Cardiovascular Ion Channel Disease Laboratory, Xi’an Jiaotong University, Xi’an 710061, People's Republic of China; d Perdana University-Royal College of Surgeons Ireland, 43400, Serdang, Selangor, Malaysia; e Institute of Cardiovascular Sciences, University of Manchester Core Technology Facility (3rd floor), University of Manchester, Manchester M13 9NT, United Kingdom

**Keywords:** Conduction defects, Multi-array recording, Genetically modified hearts, Sodium channel, Fibrosis, Brugada syndrome, Progressive cardiac conduction defect, Overlap syndrome

## Abstract

Both Brugada Syndrome (BrS) and progressive cardiac conduction defect (PCCD) are associated respectively with *diffuse* and *discrete* alterations in conduction pathways affected by ageing and sex. This study assessed for contributions of such processes to the mechanism of conduction changes in *Scn5a*^+/−^ and WT hearts stratified by age (3 and 12 months) and sex. In vivo electrocardiographic chest-lead assessment demonstrated greater incidences of bundle branch block in all *Scn5a*^*+/−*^ mice compared to WT. Frequency analysis of right ventricular (RV) epicardial activation obtained from a 64-channel multi-electrode array demonstrated greater prominence of late conducting components in *Scn5a*^*+/−*^ compared to WT male, and in male compared to female *Scn5a*^*+/−*^ following stratification by genotype and sex. Similar differences were observed between old male *Scn5a*^*+/−*^ and young male *Scn5a*^+/−^, old female *Scn5a*^+/−^, and old male WT, following stratification by genotype, age and sex. These findings directly correlated with histomorphometric assessment of regional fibrosis in both septa and free walls preferentially involving the RV. We demonstrate complex alterations in conduction distributions suggesting a conversion of normal to slow-conducting tissue, modulated by ageing and sex, coupled with fibrosis in *Scn5a*^*+/−*^ hearts. These features suggest an overlap between pathophysiological processes related to BrS and PCCD in *Scn5a*^*+/−*^ hearts.

## Highlights

► *Scn5a*^*+/−*^ hearts showed an electrocardiographic bundle branch block. ► Epicardial activation analysis demonstrated increased late conducting components. ► This paralleled a myocardial regional fibrosis preferentially involving the RV. ► Such changes were greatest in ageing and male *Scn5a*^*+/−*^ hearts. ► We propose a fibrotic mechanism slowing impulse conduction in ageing *Scn5a*^+/−^ hearts.

## 1 Introduction

A recent series of papers have explored possible associations between loss of Na^+^ channel function, age and sex, and delayed conduction changes, using the murine *Scn5a*^*+/−*^ cardiac system. These investigated their possible participation in pathophysiological changes underlying the associated arrhythmogenic syndromes. Reports from whole hearts suggested slowed conduction particularly in ageing male *Scn5a*^*+/−*^ hearts; this correlated with clinical findings in BrS patients with *SCN5A* mutations (Leoni et al., 2010; Royer et al., 2005; Stokoe et al., 2007; van Veen et al., 2005). Electrocardiographic (ECG) measurements then separated independent and interacting effects of genotype, age and sex on measures thereby demonstrating altered cardiac conduction particularly in old male *Scn5a*^*+/−*^ (Jeevaratnam et al., 2010). This in turn was related to altered dispersions of myocardial activation despite normal epicardial repolarization characteristics (Jeevaratnam et al., 2011).

Such *SCN5A* haploinsufficiencies resulting in loss of cardiac Na^+^ channel function have been associated with increased ventricular arrhythmogenic risk predisposing to sudden cardiac death. These occur in conjunction with two major clinical arrhythmic conditions. Firstly, loss of Na^+^ channel function has been implicated in 20–30% of cases of Brugada Syndrome (BrS) (Priori et al., 2000). This condition typically presents as syncope and cardiac arrest in the third or fourth decade of life. Its prevalence has been estimated at 1–5 in 10,000 worldwide (Hermida et al., 2000), with greater male (9.5%) than female (3.8%) incidences (Sacher et al., 2008). It is the leading cause of death of men under the age of 50 in endemic regions (Antzelevitch, 2002). BrS is associated with electrocardiographic right precordial ST segment elevation and complete or incomplete bundle branch block (Brugada and Brugada, 1992), often less prominent in females (Sacher et al., 2008). Arrhythmogenesis in BrS has been attributed to either depolarisation disorders delaying conduction or repolarisation disorders producing heterogeneous, epicardial action potential shortening often most prominent in the right ventricle (Wilde et al., 2010). Secondly, *SCN5A* mutations are also associated with progressive cardiac conduction defect (PCCD), also known as Lev–Lenegre disease, in some patient populations. Its epidemiological incidence has not been fully established, but it is also associated with ageing. It is clinically associated with ECG changes of either left or right bundle branch block and widening QRS complexes (Schott et al., 1999).

These conditions are associated with contrasting morphological changes. Endomyocardial biopsies in BrS patients have shown a diffuse inflammatory myocarditis, fatty tissue infiltration, interstitial fibrosis and myocyte disorganization (Frustaci et al., 2005; Ohkubo et al., 2010). These in turn may account for late potentials in signal-averaged ECGs as well as the delayed and fragmented potentials recorded from the right ventricular outflow tract (Ohkubo et al., 2010). In contrast, PCCD often manifests as discrete fibrosclerotic changes particularly affecting conduction fibres (Schott et al., 1999). There is also the possibility that these apparently contrasting features might overlap.

Our previous multi-array analysis of conduction processes in WT and *Scn5a*^*+/−*^ hearts had reported increased overall dispersion measures in conduction velocities in the old male *Scn5a*^*+/−*^ compared to the corresponding young male and old female *Scn5a*^*+/−*^ as well as old male WTs (Jeevaratnam et al., 2011). These suggested interacting effects of age, sex and genotype in compromising conduction. However, these measures did not identify whether such conduction changes reflected diffuse or discrete changes in conducting pathways. These might then correspond to the respective changes in the BrS and PCCD variants, or to possible overlaps between these disease entities.

The present paper extends those earlier studies to consider the latter question. They explore the extent to which conduction changes in the murine *Scn5a*^*+/−*^ system, and their variation with age and sex reflects diffuse, discrete or overlapping conduction changes. This involved full characterization of activation time distribution patterns obtained under different conditions systematically stratified by genotype, age and sex, for the first time. In doing so they identified discrete conducting components contributing to either early or late activation times compatible with contributions consistent with a PCCD-like change.

## 2 Methods

### 2.1 Experimental groups

Mice were housed in an animal facility at 21 °C with 12 h light/dark cycles. Animals were fed sterile chow (RM3 Maintenance Diet, SDS, Witham, Essex, UK) and had free access to water. All procedures complied with UK Home Office regulations (Animal (Scientific Procedures) Act 1986). In order to develop a comprehensive risk assessment model of the two types of mice studied, a total of 23 wild type (WT) and 21 *Scn5a*^*+/−*^ knockout mice were used. All mice used for this study were derived from their respective 129/sv background strains to avoid any possible strain-related variation in the study. The mice were divided into eight groups. Group 1 consisted of WT males aged 3 months (*n* = 7), Group 2 consisted of WT male aged >12 months (*n* = 5), Group 3 consisted of WT females aged 3 months (*n* = 6), Group 4 consisted of WT females aged >12 months (*n* = 5), Group 5 consisted of *Scn5a*^*+/−*^ males aged 3 months (*n* = 6), Group 6 consisted of *Scn5a*^*+/−*^ males aged >12 months (*n* = 5), Group 7 consisted of *Scn5a*^*+/−*^ females aged 3 months (*n* = 5) and Group 8 consisted of *Scn5a*^*+/−*^ females aged >12 months (*n* = 5). The experiments used a Langendorff-perfused preparation adapted for the murine heart, as described previously (Guzadhur et al., 2010; Stokoe et al., 2007). All assessments were performed fully blinded and codes broken only after all measurements had been performed. Mice were anesthetized with tribromoethanol (Avertin, Sigma) 240 mg/kg at a dose rate of 0.1 ml/10 g body weight. Injection was given intraperitoneally with a 27G hypodermic needle into the left peritoneal cavity. Tribromoethanol was selected in view of its less marked cardiovascular effects in comparison to other frequently used anaesthetic agents such as pentobarbital and ketamine–xylazine (Chu et al., 2006; Hart et al., 2001; Kiatchoosakun et al., 2001). Furthermore, the same anaesthetic was consistently used in all the groups of hearts studied. The mice were tilted downwards (∼30°) to ensure that visceral organs were avoided during drug injection. After injection the mice were placed in a dark cardboard cage and not disturbed till the animal was under deep sedation. For ECG recording, mice were placed in supine position on a heated platform to maintain body temperature at 37 °C. Small strips of adhesive tapes were attached to the limbs to reduce any small movement thus reducing artefacts in ECG recordings. Two 2-mm needle electrodes (MLA1204 ADInstruments, Colorado Springs, Colorado, USA) were inserted: one just above the sternum and the other into the right forelimb to produce chest lead recordings. Electrodes were then connected to a similar system as previously described (Jeevaratnam et al., 2011). The existence of bundle branch block was detected by the presence of fragmented QRS complexes in chest lead recordings.

### 2.2 Multi-electrode array recording

A custom-made electrode array consisting of 64 separate electrodes (Teflon-coated silver wires; 0.125 mm diameter; Science Products) in an 8 × 8 configuration with an inter-electrode distance of 0.55 mm was used. Unipolar recordings were performed by placing the multi-electrode array on the right epicardium with the reference electrode, acting as the indifferent pole, at the base of the heart. The 64 recording electrodes were connected through shielded wires to two 32-channel amplifiers (SCXI-1102C, National Instruments Corporation (U.K.) Ltd, Newbury, UK). The sampling frequency for each channel was set at 1 kHz. The signals were continuously stored on disk and displayed on screen using a custom-developed program, written in Labview 7.0 (National Instruments Corporation (U.K.) Ltd, Newbury, UK). For the off-line analysis, signals were displayed on screen in sets of 8–16 electrograms as previously reported (Jeevaratnam et al., 2011). The activation time was determined as the point of maximal negative slope and marked with a cursor. After marking all significant waveforms in all leads, the activation times were then displayed in a grid representing the layout of the original recording array. All activation times, in milliseconds, were related to the timing of the first detected waveform. Activation maps were drawn using Microsoft Office Excel 2007 (Microsoft Corporation, Silicon Valley, US). In order to assess distributions of activation times, the number of counts for each activation time from all hearts in their respective groups over five consecutive beats was taken and averaged. A distribution graph was plotted and the general observation of these distributions revealed the presence of two peaks and thus 6 ms was chosen as the point of separation between early and late activating components. These were then quantified by calculating the area under the graph using Origin Pro v7.5 (OriginLab Corporation, Massachusetts, USA) for each group and tested for significance in relation to other groups. The value for area under the graph in each phase represents the number of channels activating, subsequently enabling comparison between the groups.

### 2.3 Quantification of cardiac fibrosis

**Fig. 1:**
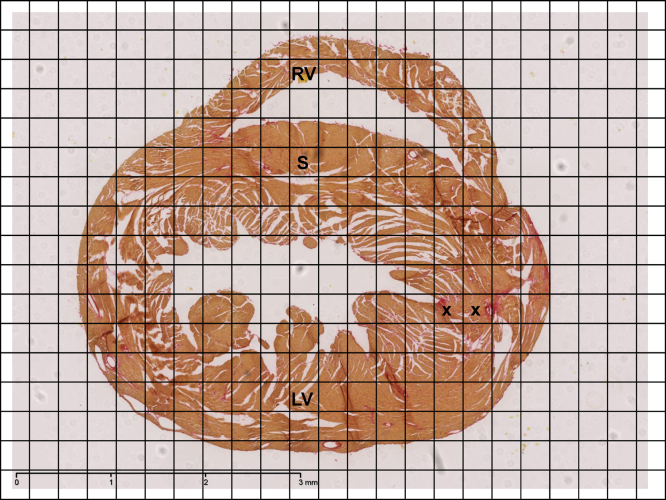
Representative slide for morphometric analysis of cardiac fibrosis at 2× magnification. Hearts were routinely stained with picrosirius red and morphometric analysis for percentage of fibrosis performed for all eight groups. Areas of increased red uptake marked by ‘x’ signify presence of fibrotic changes. The three regions were identified as represented in the figure: left ventricle (LV), septum (S) and right ventricle (RV). (For interpretation of the references to color in figure legend, the reader is referred to the web version of the article.)

Isolated hearts were flushed with Krebs buffer and perfused with 4% buffered formalin for five minutes before being immersed in formalin overnight. After fixation, gross transverse sections were cut from base to apex and the hearts were subjected to routine tissue processing and paraffin embedding. Paraffin sections of 7 μm thickness were then cut and stained with picrosirius red stain (Sigma–Aldrich, Dorset, UK). All sections were subsequently viewed, magnified and digitally acquired using the Nano Zoomer 2.0 Digital Pathology system (Hamamatsu, Hertfordshire, UK). For the quantification of fibrosis two randomly selected photomicrographs with a minimum distance of 14 μm from each other were taken for every heart. Following 2× magnification, as shown in Fig. 1, a custom made 17 cm × 23 cm morphometric grid, consisting of square boxes of dimension 1 cm × 1 cm, corresponding to a 0.26 mm × 0.26 mm area of tissue, was then superimposed on each photomicrograph. The heart was divided into three regions: left and right ventricles and septum, and the number of squares occupied by these regions counted on each photomicrograph. The number of squares containing fibrotic tissue (either partially or completely) in each region was then counted. This enabled fibrotic change to be expressed as a percentage of the cardiac tissue area. An average percentage of fibrotic changes for each region for every heart taken from the two photomicrographs was obtained and then accumulated for every group to provide the mean percentage of fibrotic tissue in each region.

### 2.4 Statistical analysis

Comparisons where appropriate were performed by Fisher's exact test or multiple analysis of variance using SPSS software (SPSS UK, Woking, Surrey). Statistical significance levels were provided, corresponding to *P* < 0.05, *P* < 0.01 or *P* < 0.001, where applicable.

## 3 Results

The experiments first investigated for gross conduction abnormalities in the murine model detectable by chest lead ECG recordings assessing for bundle branch block in the *Scn5a*^*+/−*^ mice. They then sought correlates for such changes at the tissue level through multi-electrode recordings of epicardial activation to examine for fragmentations in the distribution patterns of activation. These findings were finally correlated with the presence or absence of fibrotic changes in left and right ventricular and septal regions of the heart.

### 3.1 Electrocardiographic evidence for increased dispersions in conduction

**Fig. 2:**
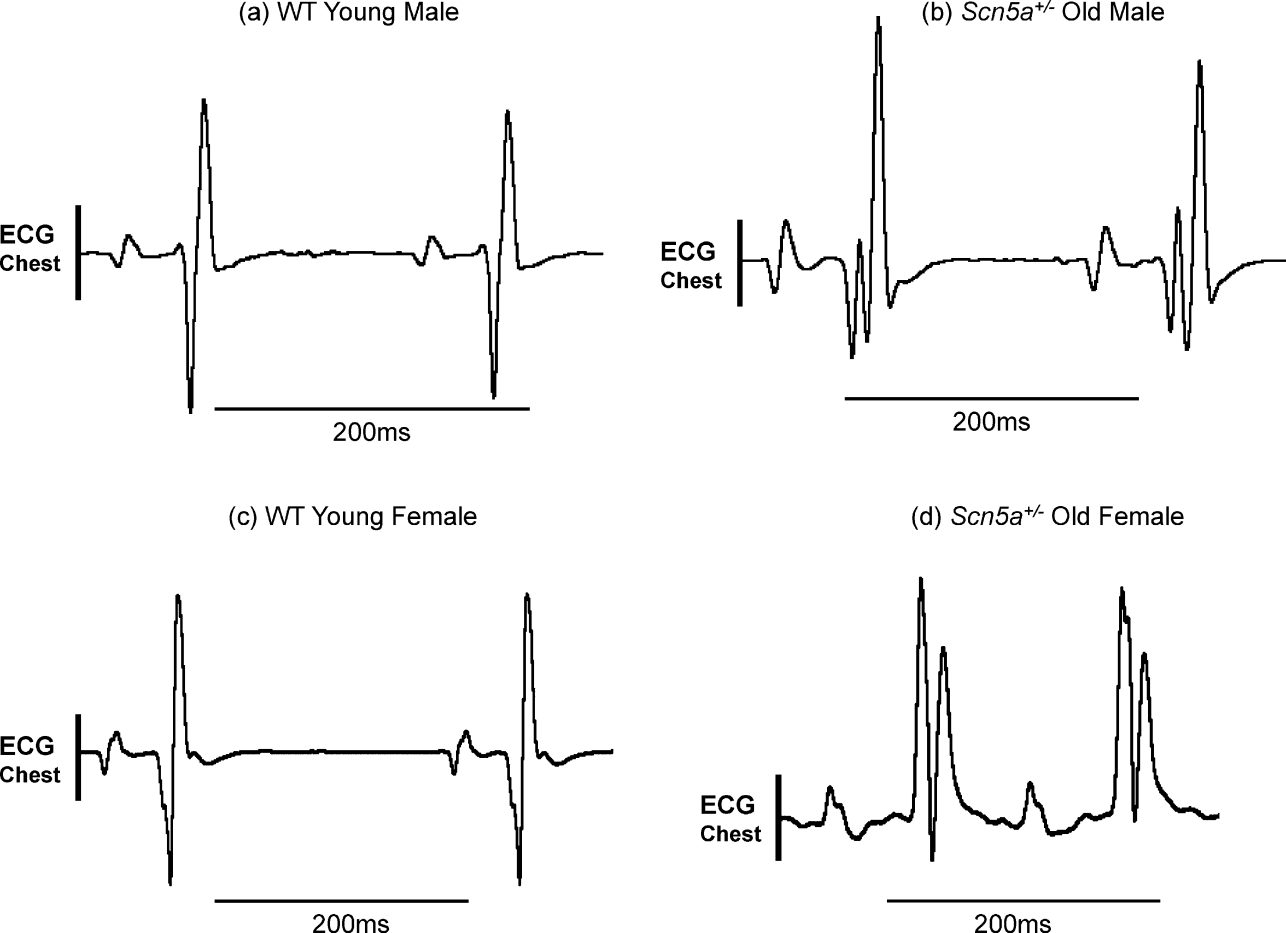
Representative ECG recordings taken from chest leads. Representative ECG recordings from young male WT (a), old male *Scn5a*^+/−^ (b), young female WT (c) and old female *Scn5a*^+/−^ (d). Chest lead recordings were obtained from intact anaesthetized mice from all the experimental groups. This demonstrated patterns of fragmented QRS complexes indicating bundle branch block that was most frequently observed in the *Scn5a*^+/−^ population.

The electrocardiographic (ECG) records of the kind shown in Fig. 2 were strongly suggestive of a fragmentation of conduction components. The ECG records, exemplified in Fig. 2 were obtained using chest leads, as opposed to the limb lead recordings obtained on the earlier occasion (Jeevaratnam et al., 2010) in anaesthetized, male (a, b) or female (c, d), young (a, c) or old (b, d), WT (a, c) and *Scn5a*^*+/−*^ mice (b, d) stratified by sex and age. This permitted a closer examination of QRS complexes in the ECGs of individual hearts. Thus the QRS complex in young male (a) or female WT mice (c) showed a simple biphasic waveform. In contrast, the QRS complex in old male (b) and female *Scn5a*^*+/−*^ mice (d) showed a considerably more complex waveform suggestive of bundle branch block. Table 1 illustrates the results of such an assessment for evidence of conduction changes in all the experimental groups and a statistical analysis for differences between such results using Fisher's exact test. These were applied to successive stratifications of the experimental groups by genotype, genotype and age, genotype and sex, and finally, genotype, age and sex. This demonstrated significantly greater incidences of traces suggestive of bundle branch block in *Scn5a*^+/−^ mice compared to wild types throughout all the four levels of stratification. In contrast, there were no significant differences between groups within populations of either WT or *Scn5a*^+/−^.

### 3.2 Multi-electrode array recordings

**Fig. 3:**
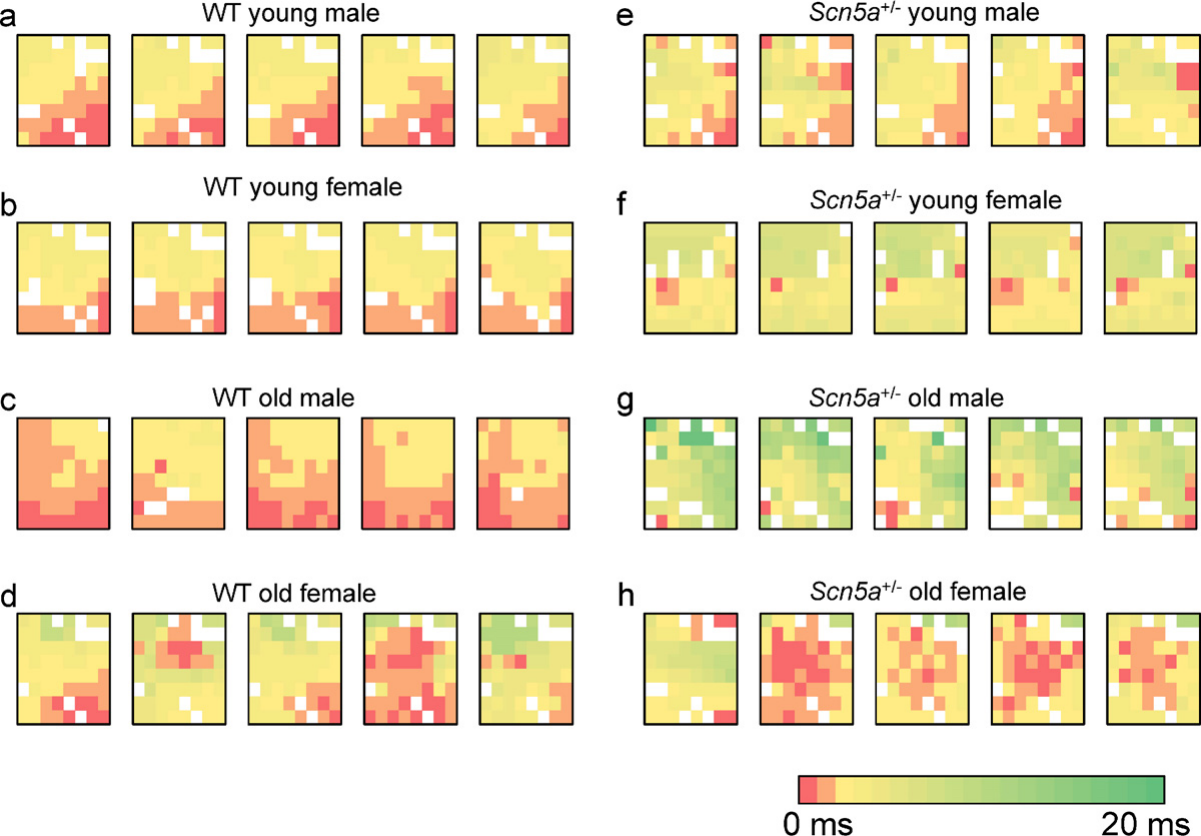
Representative activation maps of five successive cardiac cycles in WT and *Scn5a*^+/−^ hearts grouped by age and sex. The study population was stratified into: young male WT (a), young female WT (b), old male WT (c), old female WT (d), young male *Scn5a*^+/−^ (e), young female *Scn5a*^+/−^ (f), old male *Scn5a*^+/−^ (g) and old female *Scn5a*^+/−^ (h). Recordings using a 64-channel multi-electrode array on intrinsically beating Langendorff-perfused mouse hearts were performed. Activation times for 64 channels were determined in five successive cardiac cycles in all hearts to produce the respective activation maps. Colour gradients represent the various activation time for every channel. Time of first activation is represented as red followed by gradual colour change to green representing time of last activation. (For interpretation of the references to color in figure legend, the reader is referred to the web version of the article.)

Such findings at the whole heart level prompted an investigation for conduction non-uniformities at the tissue level. A 64 channel multi-electrode array recorded activation events at closely incremented (550 μm) recording sites in the RV of isolated Langendorff-perfused preparations during intrinsic activity. Biphasic recordings obtained were analysed as previously described (Jeevaratnam et al., 2011). Such latencies could then be used to construct activation maps for each cardiac event in each heart. Fig. 3 exemplifies such activation maps obtained from WT (a–d) and *Scn5a*^+/−^ (e–h), young (a, b, e, f) and old (c, d, g, h) male (a, c, e, g) and female (b, d, f, h) hearts. These resulting maps demonstrated heterogeneous patterns that included areas of particularly delayed activation. The latter were most prominent in hearts from the *Scn5a*^+/−^ mice particularly in the old mice. This was clear from increased areas showing the relevant false colours in the *Scn5a*^+/−^ maps compared to the WT mouse maps.

### 3.3 Activation time distributions

Observations of the kind outlined above were quantified by counting the total number of activated channels showing particular activation times for each single heart over five consecutive beats. This was repeated for each recording site for all the hearts in each group. The results were used to construct activation distribution curves plotting the frequency (means ± SEMs) with which individual recording sites showed particular activation times. This provided a detailed description of the distribution of activation times on the right ventricular epicardial surface in the region of interest examined.

**Fig. 4:**
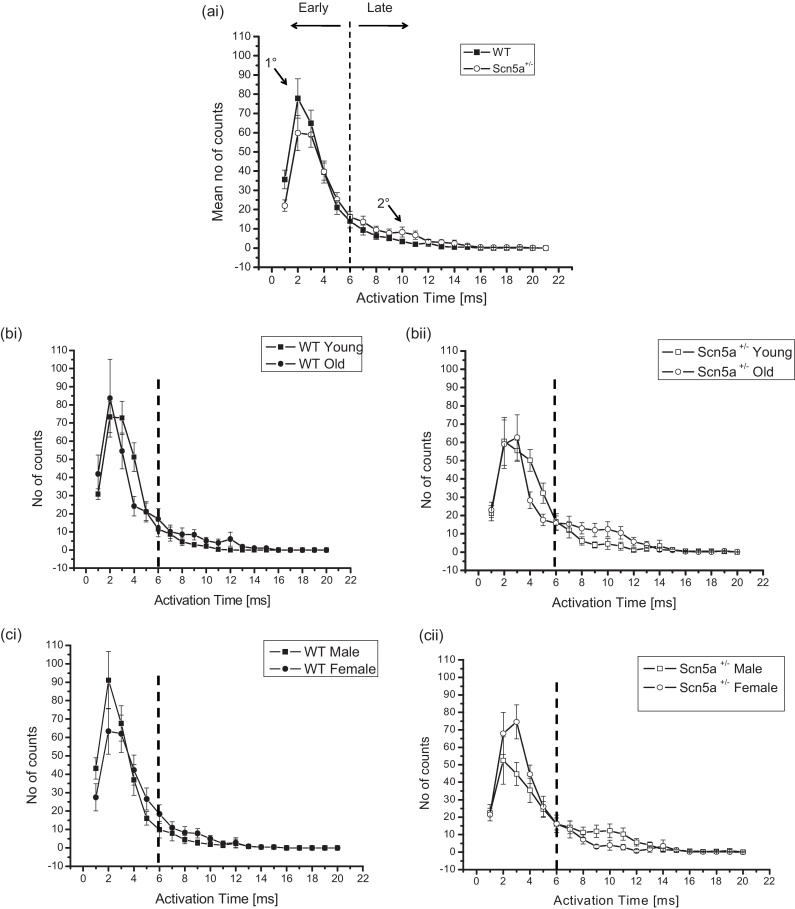
Determination of early and late activating components quantified by taking the relevant areas under the graph. The frequency distributions plot the number of channels (mean ± SEM) activating over the observed activation times in the WT and *Scn5a*^*+/−*^ population (ai) to demonstrate the separation between early and late components. The second and third levels of stratification are shown in the remaining panels, where comparisons are made between young WT and old WT (bi), young *Scn5a*^*+/−*^ and old *Scn5a*^*+/−*^ (bii), male WT and female WT (ci), and male *Scn5a*^*+/−*^ and female *Scn5a*^*+/−*^ (cii).

**Fig. 5:**
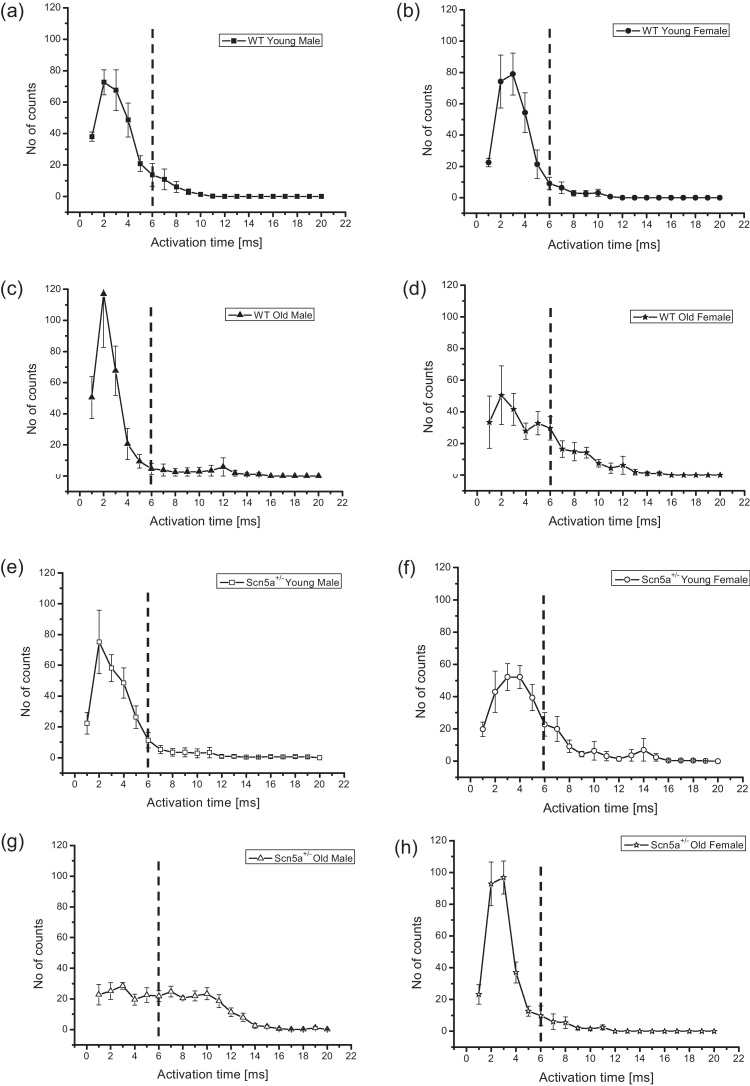
Determinations of early and late activating components quantified by taking the relevant areas under the graph. Frequency distributions for the final level of stratification: young male WT (a), young female WT (b), old male WT (c), old female WT (d), young male *Scn5a*^*+/−*^ (e), young female *Scn5a*^*+/−*^ (f), old male *Scn5a*^*+/−*^ (g) and old female *Scn5a*^*+/−*^ (h).

Fig. 4 thus summarizes the frequency distributions plotting the numbers of channels (mean ± SEM) activating over the observed activation times at different levels of stratification, in order to separate the effects of each of the factors examined. Results are therefore shown for WT and *Scn5a*^*+/−*^ (ai), young WT and old WT (bi), young *Scn5a*^*+/−*^ and old *Scn5a*^*+/−*^ (bii), male WT and female WT (ci), and male *Scn5a*^*+/−*^ and female *Scn5a*^*+/−*^ (cii). It was therefore possible to examine the effects of genotype alone, independently of age and sex, and the effects of either age or sex by themselves as variates, within a given genotype. Fig. 5 then proceeds to closer stratifications into young male WT (a), young female WT (b), old male WT (c), old female WT (d), young male *Scn5a*^*+/−*^ (e), young female *Scn5a*^*+/−*^ (f), old male *Scn5a*^*+/−*^ (g) and old female *Scn5a*^*+/−*^ (h). This permitted statistical explorations for the presence or absence of interacting effects of genotype, age and sex. An overall inspection of the resulting data set suggested a principal peak in the distribution at around 0–4 ms (labelled in Fig. 4 (ai) as “1°”) and a smaller secondary peak (labelled in Fig. 4 (ai) as “2°”) at around 8–12 ms. This bi-modal pattern of distribution was consistent with an existence of two distinct, relatively early and late classes of activation time.

Their relative contributions to the distribution was quantified to obtain the areas beneath the functions within the limits 0–6 ms and >6 ms respectively, and marked in the graphs by the dotted ordinate. This permitted quantification of the relative contributions made to the overall distribution by recording sites with the larger and smaller activation times, and of any differences in these between experimental groups generated by the different stratifications. Table 2 demonstrates that there were no differences in the relative contributions of early or late activation times either with overall stratification by genotype, or by both genotype and age. However, with stratification by genotype and sex, male *Scn5a*^+/−^ had significantly smaller early and larger late activation components compared to male WT. This difference was not shown by the female WT and female *Scn5a*^+/−^. Comparisons within the *Scn5a*^+/−^ genotypic group showed that female *Scn5a*^+/−^ mice had a larger early activation component than male *Scn5a* ^+/−^ mice. In contrast, WT hearts did not show such differences.

The final level of stratification examined for interacting differences between genotype, age and sex. This demonstrated that young and old, male and female, *Scn5a*^*+/−*^ and WT all showed indistinguishable contributions from early and late activation components. In contrast, comparisons across genotypic groups demonstrated that old male *Scn5a*^*+/−*^ hearts showed significantly smaller contributions from early activation components and significantly larger late activation components than the old male WT. However this difference was reversed in the old female *Scn5a*^*+/−*^ and WT specifically for the early activating component. Thus, the old female *Scn5a*^*+/−*^ showed significantly greater contributions from early activation components but statistically indistinguishable contributions from the late activation components compared to the old female WT. Furthermore, comparisons within genotypic groups revealed that the old male *Scn5a*^*+/−*^ showed significantly smaller contributions from early activation components and a significantly larger contribution from late activation components compared to both the young male *Scn5a*^*+/−*^ and old female *Scn5a*^*+/−*^ mice. This is in contrast to findings observed in the female WT. Thus, the old female WT had a significantly smaller contribution from early activation components and a significantly larger contribution from late activation components than the young female WT.

Our study, importantly also observed that total activating components (Table 2) which represent the cumulative area for both early and late activating components were unchanged in all levels of stratifications except when comparing between *Scn5a*^*+/−*^ male and female. Together, they demonstrate that while the total availability of conducting components remain unchanged, a sub-population of these existing components are altered, giving rise to a distinct delayed conducting component.

### 3.4 Assessments of fibrotic change

The electrophysiological experiments above demonstrated early and late activation components mediating myocardial conduction and redistributions in their contributions that were attributable to genotype, age and sex. We next sought correlations between these changes and the presence and extent of fibrotic change in the left and right ventricular free walls and septum with similarly stratified groups of picrosirius red stained hearts. Table 3 demonstrates that stratification solely by genotype showed that *Scn5a*^*+/−*^ hearts had significantly more fibrotic tissue in all regions studied compared to WT. Stratifications by age then demonstrated greater fibrosis in old than young WT left ventricle and septum, and in old than young *Scn5a*^+/−^ septum. Nevertheless, both young and old *Scn5a*^+/−^ showed more fibrosis than the corresponding WT in all regions. Stratification by sex indicated indistinguishable levels of fibrosis between WT male and WT females in all regions. In contrast, *Scn5a*^+/−^ males showed greater fibrosis than *Scn5a*^+/−^ females specifically in the left ventricle. Similarly, both male and female *Scn5a*^+/−^ showed more fibrosis compared to corresponding male and female WT in all regions apart from the left ventricles of *Scn5a*^+/−^ females and WT females. Full stratifications into genotype, age and sex demonstrated that old male *Scn5a*^*+/−*^ showed more fibrosis than young male *Scn5a*^*+/−*^, old female *Scn5a*^*+/−*^ and old male WT in all regions. In contrast old male WT showed more fibrosis than young male WT in the left ventricle and septa, but similar fibrosis levels as old female WT. Comparisons of comparable groups between *Scn5a*^*+/−*^ and WT revealed increased fibrosis in the *Scn5a*^*+/−*^ groups in all the regions assessed apart from in old females. Finally, ratios between levels of fibrosis in the *Scn5a*^+/−^ compared to WT hearts were systematically greater in the right ventricle compared to other regions (Table 4). These results together suggest an increased fibrosis resulting from the *Scn5a*^*+/−*^ mutation, exacerbated in males and by ageing; this involved all cardiac regions, but more so in the RV, in direct corroboration of the electrophysiological results.

## 4 Discussion

The present study concludes a series of reports describing independent and interacting effects on action potential conduction of the *Scn5a*^*+/−*^ genotype with age and sex using systematically stratified experimental groups and comparing these with fibrotic change. These final experiments were prompted by reports that *SCN5A* mutations are associated with not only with Brugada Syndrome (BrS), but also with progressive cardiac conduction defect (PCCD)/Lev–Lenegre disease. These conditions are accompanied by differing morphological changes. They compared features in the genetically modified murine *Scn5a*^*+/−*^ model with correspondingly matched WT hearts, at the levels of electrocardiographic recording, a frequency distribution analysis of multi-electrode array recordings, and histological assessments of regional tissue fibrosis. They add to previous reports (Jeevaratnam et al., 2010, 2011) on the effect of age, sex and BrS genotype upon action potential conduction in the following respects.

First, they used electrocardiographic chest rather than the limb lead recordings employed on earlier occasions (Jeevaratnam et al., 2010, 2011). The results could then be compared with the clinical findings of right chest lead ST segment elevation and bundle branch block used to diagnose BrS. They demonstrate a correspondingly high incidence of such features in the murine *Scn5a*^+/−^ model for the first time

Secondly, previous reports (Jeevaratnam et al., 2011) provided quantitative indicators for overall spatial and temporal dispersions of activation. However they did not discriminate between *diffuse* alterations in conduction properties, *discrete* alterations in particular conducting fibres, or whether both features co-existed in an overlap syndrome. On the one hand, endomyocardial biopsies in BrS patients have shown *diffuse* inflammatory myocarditis, fatty tissue infiltration, interstitial fibrosis and myocyte disorganization (Frustaci et al., 2005; Ohkubo et al., 2010). These may account for late potentials in signal-averaged ECGs as well as the delayed and fragmented potentials recorded from the right ventricular outflow tract (Ohkubo et al., 2010). On the other hand, PCCD often manifests as *discrete* fibrosclerotic changes particularly affecting conduction fibres (Schott et al., 1999). Its epidemiological incidence has not been fully established, but it is also associated with ageing. It is clinically associated with ECG changes of either left or right bundle branch block accompanied by widening QRS complexes (Schott et al., 1999).

The present studies extended the previous analysis (Jeevaratnam et al., 2011) to obtaining and comparing detailed frequency distributions representing dispersions of conduction times, between experimental groups. They preserved the population stratification adopted in the previous paper and could therefore relate particular findings to age and sex. They thus distinguished between the two possible mechanisms for observed conduction changes indicated above. *Diffuse* alterations in conduction properties would predict a single frequency distribution of activation times, or frequency distributions that would not vary in relative magnitude between experimental groups, but whose position would differ between experimental groups. In contrast, the present studies demonstrated *discrete* early and late activation components from accordingly bimodal rather than unimodal frequency histograms, consistent with discrete populations of relatively rapidly and slowly conducting pathways. They then characterized these distinct relative contributions to overall conduction properties in the different experimental groups for the first time. This showed clear shifts in such distributions from the early to the late activating conduction components in the distributions, particularly in *Scn5a*^+/−^ hearts especially in old male mice. In contrast, there were no shifts in their peaks. These differences took place despite similar cumulative areas beneath the histograms covering both the early and late activating components at all stratification levels apart from those comparing male and female *Scn5a*^*+/−*^. Together these findings suggest that the alterations in relative contributions took place despite an unchanged total availability of conducting components.

Thirdly, the present experiments extended previous anatomical studies reporting right ventricular fibrotic changes in *Scn5a*^+/−^ mice (Jeevaratnam et al., 2011), by a histological examination of the free walls of both ventricular chambers as well as the septa, stratified similarly as the electrophysiological studies. This confirmed fibrotic changes preferentially affecting the right ventricle. This parallels clinical reports describing structural abnormalities such as fibrosis and fatty infiltration, electrocardiographic ST segment elevations and a compromised local activation in basal RV epicardium (Hoogendijk et al., 2010b). However such changes also involved the left ventricle and septum additionally implicating bilateral conduction defects and sclerotic lesions in the Purkinje system and bundle branches in both chambers as found in PCCD.

Together the present findings thus demonstrate for the first time bimodal distribution patterns in frequency histograms describing conduction in murine *Scn5a*^+/−^ hearts. They attributed the observed conduction patterns to redistributions between discrete rapid and slow conducting components rather than diffuse shifts in such conduction peaks, with genotype, ageing and sex exerting independent and interacting effects. Such changes might also cause the bundle branch block observed in chest lead recordings at the whole animal level. These findings correlated directly with fibrotic changes that, whilst most prominent in the RV, affected all ventricular myocardial regions, in parallel with an overlapping PCCD-like change. They suggest a progressive conduction defect associated with the development of discrete structural changes converting normal to slow-conducting tissue particularly in old male *Scn5a*^*+/−*^ hearts.

The present findings integrate previous reports separately associating arrhythmic patterns resembling human BrS (Stokoe et al., 2007) or fibrotic PCCD-like change with this particular loss-of-function mutation model (Jeevaratnam et al., 2010, 2011; Leoni et al., 2010; Royer et al., 2005; van Veen et al., 2005). They also recapitulate recent clinical reports of impaired cardiac conduction in BrS probands carrying altered *SCN5A* as opposed to BrS patients that did not carry such abnormal *SCN5A* (Smits et al., 2002). They also support suggestions that the most common phenotype in BrS-type *SCN5A* mutation carriers is a progressive conduction defect indistinguishable from that reported in PCCD. Such fibrosis might account not only for delayed conduction but also for magnetic resonance imaging observations of right ventricular outflow tract dilatation in BrS (Catalano et al., 2009). There is therefore the possibility that the reduced conduction leading to arrhythmogenic tendency in BrS requires a *combination* of altered Na^+^ channel expression and tissue structural change, rather than either factor alone. Certainly recent computer modeling studies reported that reduced sodium current only hinders activation when accompanied by structural abnormalities suggesting such an interaction of these factors (Hoogendijk et al., 2010a, 2010b).

## Figures and Tables

**Table 1:** Incidence of hearts with ECG traces suggesting bundle branch block.

Animal	*n*	Number of hearts		Animal	*n*	Number of Hearts	
		Present	Absent			Present	Absent
WT (TOTAL)^μ^	23	0	23	*Scn5a*^*+/−*^ (TOTAL)^μ^	20	16	4
WT young^β^	11	0	11	*Scn5a*^*+/−*^ young^β^	9	8	1
WT old^ω^	12	0	12	*Scn5a*^*+/−*^old^ω^	11	8	3
WT male^λ^	12	0	12	*Scn5a*^*+/−*^ male^λ^	12	9	3
WT female^Ω^	11	0	11	*Scn5a*^*+/−*^ female^Ω^	8	7	1
WT young male^*^	6	0	6	*Scn5a*^*+/−*^young male^*^	6	5	1
WT young female^φ^	5	0	5	*Scn5a*^*+/−*^ young female^φ^	3	3	0
WT old male^‡^	6	0	6	*Scn5a*^*+/−*^ old male^‡^	6	4	2
WT old female^α^	6	0	6	*Scn5a*^*+/−*^ old female^α^	5	4	1

Values with similar superscript differ significantly with each other at *P *< 0.05. *P*: ^μ^<0.001, ^β^<0.001, ^ω^<0.001, ^λ^<0.001, ^Ω^<0.001, ^*^0.008, ^φ^0.018, ^‡^0.030, ^α^0.015.

**Table 2:** Area under the graph.

Animal	*n*	Area (Mean ± SEM)			Animal	*n*	Area (Mean+SEM)		
		Total	EAC	LAC			Total	EAC	LAC
WT	23	265.57 ± 4.49	239.76 ± 7.62	25.80 ± 7.71	*Scn5a*^*+/−*^	21	268.10 ± 4.32	217.79 ± 13.81	50.31 ± 12.19
WT young	13	264.04 ± 3.52	249.81 ± 7.57	14.23 ± 5.90	*Scn5a*^*+/−*^ young	11	265.05 ± 6.97	231.59 ± 16.58	33.45 ± 14.33
WT old	10	267.55 ± 9.55	226.70 ± 13.91	40.85 ± 15.15	*Scn5a*^*+/−*^ old	10	271.45 ± 5.02	202.60 ± 22.46	68.85 ± 19.21
WT male	12	265.83 ± 7.52	247.13 ± 11.45^μ^	18.71 ± 10.34^α^	*Scn5a*^*+/−*^ male	11	258.86 ± 5.45^a^	191.64 ± 20.28^b,μ^	67.23 ± 18.96^α^
WT female	11	265.27 ± 4.98	231.72 ± 9.88	33.55 ± 11.56	*Scn5a*^*+/−*^ female	10	278.25 ± 5.38^a^	246.55 ± 14.55^b^	31.70 ± 13.42
WT young male	7	263.57 ± 4.81	247.71 ± 11.74	15.86 ± 9.36	*Scn5a*^*+/−*^ young male	6	253.83 ± 9.20	233.33 ± 24.48^g^	20.50 ± 16.48^h^
WT young female	6	264.58 ± 5.63	252.25 ± 10.16^e^	12.33 ± 7.56^f^	*Scn5a*^*+/−*^ young female	5	278.50 ± 7.57	229.50 ± 24.73	49.00 ± 24.72
WT old male	5	269.00 ± 17.91	246.30 ± 23.98^β^	22.70 ± 22.7^δ^	*Scn5a*^*+/−*^ old male	5	264.90 ± 4.41	141.60 ± 14.66^c,g,β^	123.30 ± 11.36^d,h,δ^
WT old female	5	266.10 ± 9.41	207.10 ± 10.19^e,¥^	59.00 ± 18.78^f^	*Scn5a*^*+/−*^ old female	5	278.00 ± 8.52	263.60 ± 13.98^c,¥^	14.40 ± 7.07^d^

Values with similar superscript differ significantly with each other at *P* < 0.05. *P*: ^a^0.021, ^b^0.044, ^c^<0.001, ^d^<0.001, ^e^0.013, ^f^0.036, ^g^0.014, ^h^0.001, ^μ^0.024, ^α^0.032, ^β^0.006, ^δ^0.004, ^¥^0.011. EAC: Early activating component, LAC: Late activating component.

**Table 3:** Percentage (%) of fibrotic tissue in murine hearts.

Animal	*n*	Left ventricle	Septum	Right ventricle	Animal	*n*	Left ventricle	Septum	Right ventricle
WT	20	5.83 ± 1.08^¥^	5.03 ± 0.95^θ^	1.86 ± 0.56^П^	*Scn5a*^*+/−*^	17	14.96 ± 2.26^¥^	18.70 ± 3.40^θ^	10.41 ± 1.62^П^
WT young	10	2.44 ± 0.39^a,ψ^	2.42 ± 0.52^b,€^	1.19 ± 0.50^Ω^	*Scn5a*^*+/−*^ young	8	10.77 ± 1.28^ψ^	8.60 ± 2.06^c,€^	8.44 ± 1.72^Ω^
WT old	10	9.23 ± 1.49^a,ℓ^	7.66 ± 1.42^b,*^	2.52 ± 0.99^#^	*Scn5a*^*+/−*^ old	9	18.70 ± 3.78^ℓ^	27.68 ± 4.37^c,*^	12.17 ± 2.61^#^
WT male	10	5.10 ± 1.29^§^	5.34 ± 1.30^μ^	1.43 ± 0.95^Σ^	*Scn5a*^*+/−*^ male	10	20.19 ± 2.72^d,§^	23.96 ± 4.98^μ^	11.73 ± 2.42^Σ^
WT female	10	6.58 ± 1.77	4.73 ± 1.45^ω^	2.29 ± 0.62^†^	*Scn5a*^*+/−*^ female	7	7.51 ± 1.21^d^	11.19 ± 2.46^ω^	8.52 ± 1.86^†^
WT young male	6	2.58 ± 0.63^p,‡^	2.72 ± 0.85^q,◊^	0.83 ± 0.62^Δ^	*Scn5a*^*+/−*^ young male	4	12.42 ± 1.93^s,‡^	6.93 ± 1.91^v,◊^	5.66 ± 1.59^w,Δ^
WT young female	4	2.23 ± 0.33^r,λ^	1.97 ± 0.37	1.74 ± 0.85^¶^	*Scn5a*^*+/−*^ young female	4	9.12 ± 1.47^λ^	10.26 ± 3.78	11.21 ± 2.47^¶^
WT old male	4	8.86 ± 1.88^p,ff^	9.28 ± 1.52^q,β^	2.31 ± 1.31^α^	*Scn5a*^*+/−*^ old male	6	25.36 ± 2.69^s,x,ff^	35.31 ± 2.89^v,y,β^	15.78 ± 2.90^w,z,α^
WT old female	6	9.48 ± 2.28^r^	6.57 ± 2.14	2.66 ± 0.89	*Scn5a*^*+/−*^ old female	3	5.36 ± 1.38^x^	12.44 ± 3.50^y^	4.94 ± 0.88^z^

Values with similar superscript differ significantly with each other at *P* < 0.05. *P*: ^a^<0,001, ^b^0.003, ^c^0,002, ^d^0.002, ^p^0,006, ^q^0.003, ^r^0.035, ^s^0,008, ^v^<0,001, ^w^0,030, ^x^0.002, ^y^0,002, ^z^0,039, ^¥^0.001,^θ^<0,001, ^П^<0,001, ^ψ^<0.001, ^€^0.005, ^Ω^<0.001, ^ℓ^0.027, ^*^<0.001, ^#^0,002, ^§^<0.001, ^μ^0.002, ^ω^0,029, ^Σ^0.002, ^†^0.002, ^◊^0.011, ^Δ^0.052, ^λ^0.035,^¶^0.011, ^ff^0.002, ^β^<0.001, ^α^0.011, ^‡^<0.001.

**Table 4:** Ratio of fibrotic changes as associated with genotype.

Groups	Ratio ± SE		
	Left ventricle	Septum	Right ventricle
*Scn5a*^*+/−*^ vs WT	2.57 ± 0.61	3.72 ± 0.97	5.60 ± 1.90
*Scn5a*^*+/−*^ young vs WT young	4.41 ± 0.88	3.55 ± 1.14	7.09 ± 3.31
*Scn5a*^*+/−*^ old vs WT old	2.03 ± 0.52	3.61 ± 0.88	4.83 ± 2.16
*Scn5a*^*+/−*^ male vs WT male	3.96 ± 1.13	4.49 ± 1.44	8.20 ± 5.71
*Scn5a*^*+/−*^ female vs WT female	1.14 ± 0.36	2.37 ± 0.89	3.72 ± 1.30
*Scn5a*^*+/−*^ young male vs WT young male	4.81 ± 1.39	2.55 ± 1.06	6.82 ± 5.44
*Scn5a*^*+/−*^ young female vs WT young female	4.09 ± 0.89	5.21 ± 2.15	6.44 ± 3.45
*Scn5a*^*+/−*^ old male vs WT old male	2.86 ± 0.68	3.80 ± 0.69	6.83 ± 4.07
*Scn5a*^*+/−*^ old female vs WT old female	0.57 ± 0.24	1.89 ± 0.81	1.86 ± 0.70

No significant differences were observed between any groups.
